# Organizational Drivers of Burnout and Work Engagement: A Multilevel Study in Portuguese Firefighter Brigades

**DOI:** 10.3390/ijerph19074053

**Published:** 2022-03-29

**Authors:** Susana Llorens, Marisa Salanova, María José Chambel, Pedro Torrente, Rui P. Ângelo

**Affiliations:** 1 WANT Research Team, Universitat Jaume I, 12071 Castelló de la Plana, Spain; llorgum@uji.es (S.L.); torrente@uji.es (P.T.); 2 CICPSI, Faculdade de Psicologia, Universidade de Lisboa, 16499-013 Lisboa, Portugal; mjchambel@psicologia.ulisboa.pt (M.J.C.); ruiangelo@edu.ulisboa.pt (R.P.Â.)

**Keywords:** job demands, proactive coping, social support, burnout, work engagement

## Abstract

In this study, we analyzed how organization-level demands and organizational-level social support relate to the core dimensions of burnout and work engagement, controlling for individual resources (i.e., proactive coping) and demands (i.e., acute demands) using the Job Demands-Resources Theory. In a sample of 1487 Portuguese firefighters nested within 70 fire brigades, hierarchical linear modeling indicated that: (1) proactive coping was related to lower burnout and higher work engagement, whereas acute demands were related to higher burnout and lower work engagement (for vigor only); (2) proactive coping moderated the relationship between acute demands and vigor; and (3) unexpectedly, social support from colleagues was not related to firefighters’ well-being, whereas organization-level demands were related to higher burnout and lower work engagement. These results suggest the need to implement practices and policies to guarantee the relevant conditions for improving the well-being of firefighters, to develop coping strategies in a proactive way, and finally, to enhance support from colleagues.

## 1. Introduction

Firefighters aim to protect citizens’ lives in emergency situations. They experience threatening work conditions that influence their well-being due to cumulative exposure in their daily work life [1,2]. Fire personnel intervene in incidents that involve severe injuries, life-threatening circumstances, and death, which are potentially traumatic events [3,4,5]. Hence, firefighters are exposed to very intense stressors for short periods of time, and psychological stress is part of their daily lives and affects their well-being [1].

As one of the most widely applied theoretical models to test both burnout and work engagement, the Job Demands-Resources Model (JD-R Model) [6] differentiates job demands and job resources as the main antecedents of well-being at work. Although job demands are not necessarily negative, they become stressors leading to chronic fatigue [7,8]. This impairment process is especially consistent for burnout syndrome, as work demands may deplete employees’ energy and hinder their attachment to the organization [9,10]. In contrast, job resources are intrinsically motivating for employees, and thus, they have shown a consistent association with work engagement [9,11]. Resources are functional in achieving work goals and help to deal with work demands, thus promoting work engagement and, even more, preventing burnout, as it can be considered the opposite of work engagement [12]. 

Recent reviews on the JD-R Model call for further research on multilevel processes in order to address the complexity of organizational phenomena and deepen theoretical development [13,14,15,16]. The premise is that organizational environments tend to expose individuals to common policies, practices, and procedures [17]. In this way, people who work in the same organization tend to develop shared interpretations and perceptions [18]. Thus, some demands and resources emerge as constructs of a higher level of analysis (i.e., organizational) due to the consensus in the perceptions of the members of the same organization with an isomorphic functioning with respect to a lower level (i.e., individual) [19,20]. However, we intend to apprehend the organizational context, without disregarding the individual perceptions of those who are part of it [21]. In this way, we intend to analyze to what extent the firefighters’ well-being is affected by the demands and resources at the individual level and by the demands and resources at the organizational level. 

**Figure 1:**
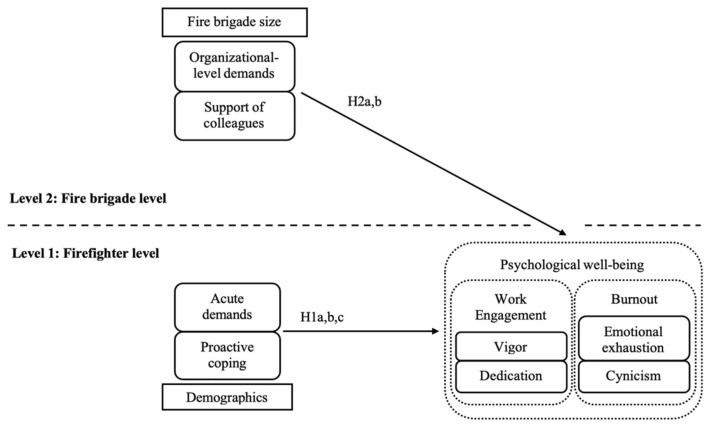
Research model involving individual firefighter-level predictors (H1a, b, c) and organizational fire brigade-level predictors (H2a, b, cross-level hypotheses).

We summarize the general model and specific hypotheses tested in this study in Figure 1. The purpose of this study is to examine how demands and resources may relate to psychological well-being in a multilevel analytical context (individual and organization). In general, we propose two levels of analyses: an individual level of analysis (acute demands and proactive coping to predict work engagement and burnout); and the cross-level influences between organization-level and individual-level variables (organizational demands and support of colleagues to predict firefighter’s well-being). The study is important since although the impact of demands and resources on well-being has been tested before in different samples across cultures [22,23,24,25,26], this is the first time we test the model considering acute and organizational level demands as well as the support of colleagues (aggregated at organizational level) and proactive coping using multilevel analyses. Furthermore, it is the first time that this theoretical model is tested in a specific sample: firefighters. The results will be useful for practitioners to enhance positive work conditions in order to generate well-being in employees, particularly in a special and relevant professional group: firefighters.

### 1.1. Burnout and Work Engagement

A dual perspective of well-being at work including negative psychological states (i.e., burnout) and positive psychological states (i.e., work engagement) contributes to a more accurate understanding of the motives and affects in job settings [12,27]. Burnout syndrome is a “prolonged response to chronic emotional and interpersonal stressors on the job, and is defined by the three dimensions of exhaustion, cynicism and professional inefficacy” [28]. On the positive side of well-being at work, work engagement is regarded as the positive antipode of burnout [29]. Firstly, conceptualized by Kahn [30], work (or employee) engagement is a “positive, work-related state of mind, that is characterized by vigor, dedication and absorption” [31]. Both burnout and work engagement have been regarded as opposing states that mediate between opposing processes leading to health impairment (i.e., burnout) and work motivation (i.e., work engagement) [10]. Furthermore, the core dimensions of burnout and work engagement remain at the opposite end of two different continua, namely, activation and identification [32]. At the activation pole, emotional exhaustion involves the depletion of emotional resources; thus, the employee feels fatigued and emotionally drained, while vigor refers to feeling persistent against difficulties as well as energetic, strong, and devoted to his/her task. The identification pole relates to cynicism and dedication. Cynicism involves the indifference of a distant attitude towards one’s job or towards whom he/she works with; while dedication refers to being emotionally attached to the task at hand, which provides the employee with a sense of meaning and purpose towards his or her work. 

Emotional exhaustion and cynicism constitute the core of burnout syndrome [33], with professional efficacy being a consequence of it [34]. As an opposite to the core dimensions of burnout, vigor and dedication are considered the core dimensions of work engagement [31], with absorption being a plausible consequence of it [32]. In the current study, we included the core dimensions of burnout (i.e., emotional exhaustion and cynicism) and work engagement (i.e., vigor and dedication). The rationale for the perspective taken in this study is two-fold. Firstly, this is coherent with the idea of offering an integrative picture of emotional states in organizations by means of including both the negative (i.e., burnout) and positive (i.e., work engagement) indicators of psychological well-being at work [34]. Secondly, based on the JD-R Model, the study provides a closer examination of the antecedents of the separate dimensions of burnout and work engagement. Considering emotional exhaustion, cynicism, vigor, and dedication as separate dependent variables addresses the call for further research and conceptual development of burnout and work engagement based on their inner core components as opposite states in the energy and identification poles [35,36,37]. Furthermore, we add to the incipient research avenue [38] on the positioning of the constructs as polar opposites or overlapping work experiences attending to differential patterns of relationships of their core dimensions with antecedents and/or consequences.

### 1.2. Acute Demands and Proactive Coping Strategies: Firefighters-Level Hypotheses

In the context of fire personnel, acute demands are “unusual situations that hinder the responsiveness of the firefighter and lead to strong emotional reactions”. In emergency and ambulatory workers, these unexpected events may result in higher stress usually combined with negative emotions and feelings of despair and poignancy [39]. A conscious effort to deal with the negative effects may result in energy depletion as the firefighter is required to actively confront these demands as part of their job [40]. As a consequence, exposure to these demands may, in the long run, lead to emotional exhaustion (i.e., feeling emotionally drained and fatigued) and less vigor (i.e., feeling less vigorous and energetic during the required tasks and missions). This provides an explanation to why employees under strain invest in recovery activities in order to achieve an adequate level of psychological detachment from work [41,42]. In a more dysfunctional process to prevent negative emotional reactions to acute demands, however, employees might develop cynical attitudes (i.e., psychological distance from one’s job or the people with whom one works) and diminish their levels of dedication (i.e., getting less enthusiastic and inspired with work). Furthermore, the individual perceptions and interpretations about these acute demands were more predictive of their response and well-being consequences than the severity of the events [43]. Therefore, we expect that:

**Hypothesis** **1a** **(H1a).** *Acute individual demands are positively related to burnout (i.e., emotional exhaustion and cynicism) and negatively related to work engagement (i.e., vigor and dedication)*.

Demanding work environments such as those experienced by fire personnel require proper strategies that contribute to their well-being. Coping strategies are cognitive or behavioral efforts carried out to cope with demands that tax or exceed the personal resources of an individual [44]. Thus, coping strategies alleviate emotional distress caused by overwhelming events that are perceived as harmful, threatening, or uncontrollable. Specifically, proactive coping strategies are defined as “an effort to build up general resources that facilitate promotion toward challenging goals and personal growth” [45]. As compared with other types of coping, a proactive coping style is focused on future events that are perceived as self-promoting, thus helping to overcome their negative consequences [46]. Examples of this type of coping strategies include arranging resources to be used in an optimal way, realistic goal setting and appraisal of future events, and effective use of performance feedback [47]. Employees that deal with recurrent and unpredictable emergency situations may benefit from making use of a proactive coping style as they may perceive themselves more capable of successfully meeting their goals [48] or may take active steps to cope with stressors on their own or with other colleagues [12]. Apart from this expected, direct positive effect over psychological well-being at work (i.e., decreasing burnout and increasing work engagement), there is empirical support for expecting an indirect, positive effect of proactive coping as a moderator of work demands. By definition, coping strategies are conscious efforts in which individuals engage when the expected circumstances overcome the resources they have at hand [49]. Hence, these strategies are oriented to reduce the impact of the demands. In the case of fire personnel, proactive coping strategies may buffer the negative effect of acute demands over firefighters’ psychological well-being. Although scarcely researched in this specific population, previous literature suggested that well-being at work may benefit (i.e., less burnout and more work engagement) from applying proactive coping strategies as a means of dealing with highly demanding situations with potential harmful effects [50,51]. Therefore, we expect that:

**Hypothesis** **1b** **(H1b).** *Proactive coping strategies are negatively related to burnout (i.e., emotional exhaustion and cynicism) and positively related to work engagement (i.e., vigor and dedication)*.

**Hypothesis** **1c** **(H1c).** *Proactive coping strategies moderate the association between acute demands and both burnout and work engagement. Therefore, when proactive coping strategies are high, the relation between acute demands and burnout (i.e., emotional exhaustion and cynicism) will become weaker, and the relation between acute demands and work engagement (i.e., vigor and dedication) will become stronger*.

### 1.3. Organizational-Level Demands and Social Support: Brigade-Level Hypotheses

Stress in fire personnel is not only caused by individual characteristics or demands at firefighter level [52]. Fire brigades consist of direct command and control hierarchies, have clear goals, and are functionally organized to fulfil their duties. Because of the high level of coordination required to complete their tasks successfully during fire emergency situations, firefighters share common experiences, values, knowledge, and perspectives to understand organizational events and interpret organizational-level demands. In this context, organizational-level demands are unit or organizational characteristics that require sustained mental effort, and hence, are associated with physiological or psychological costs [6]. Examples of firefighters’ organizational-level demands are the use of inadequate or impaired equipment or technology, understaffed situations, excessive overwork or double-shift due to lack of proper work scheduling, misleading information or wrong reports about scenarios, and coordination with other rescue forces. Along with unpredictable emergency situations, these organizational-level demands, which affect the whole fire brigade and are beyond their individual control, have consequences on their well-being [53]. Therefore, we expect that:

**Hypothesis** **2a** **(H2a).** *Organizational-level demands are positively related to burnout (i.e., emotional exhaustion and cynicism) and negatively related to work engagement (i.e., vigor and dedication)*.

Although organizational-level demands become shared stressors within the fire brigades, social support provided by colleagues, or the supervisor becomes a shared, social resource. In fact, workers in the same context may share their perceptions about the degree to which colleagues and supervisors are committed to them, as there may be a collective perception of social support [54]. Social support is a social resource as far as it is functional in achieving work goals through enabling back-up behaviors [55]; reducing job demands and the associated physiological and psychological costs since individuals provide each other with support that may reduce the strain experience and mitigate perceived stressors [56,57]; stimulating personal growth and development through nurturing social relationships [58,59]; and promoting work engagement [60,61]. The collective nature of social support was validated by different authors [54,62,63].

**Hypothesis** **2b** **(H2b).** *Social support from colleagues and social support from supervisors are negatively related to burnout (i.e., emotional exhaustion and cynicism) and positively related to work engagement (i.e., vigor and dedication)*.

## 2. Materials and Methods

### 2.1. Participants

Data were gathered by means of a protocol between the University of Lisbon—Organizational Psychology Department and the Portuguese Government’s Firefighting Agency. Altogether, 2025 rescue mission firefighters from every district in Portugal (18 in all) were invited to participate. Each firefighter received a questionnaire, instructions on how to fill it out, and return envelopes. We received 1610 questionnaires (80% response rate) and 1487 (92%) from 70 fire brigades in Portugal were complete and constitute the sample. The mean age was 35.2 years (SD = 9.1 years), 98% were male, and the average number of years’ experience was 13.7 years (SD = 8.2 years). The type of firefighter was also taken into account (39% volunteers, 38% professional, and 23% municipals). Firefighters were nested within 70 fire brigades, with a mean brigade size of 21.2 (SD = 20.6). A comparison of the sample characteristics with those of the population (in accordance with Government Agency records) revealed no differences in age, gender or years of firefighting experience. The study was approved by the Ethics Committee of Faculty of Psychology, University of Lisbon and by the Institutional Review Board (or Ethics Committee) of Universitat Jaume I.

### 2.2. Measurement Instruments

Individual Level. Acute demands were measured through the respective six items of Portuguese Rescue Mission Firefighters—Professional Demands Scale [39], (e.g., ‘Scenarios of multi-trauma-3 or more victims with serious injuries’; α = 0.74). Items were scored on a five-point scale ranging from 1 (rarely) to 5 (very often).

Proactive coping was measured with five items (e.g., ‘After attaining a goal, I look for another, more challenging one’; α = 0.71) from the Proactive Coping Inventory [64]. Participants were asked to indicate the extent to which each statement characterizes them on a four-point scale ranging from 0 (never) to 4 (always).

Organizational Level. Organization-level demands were measured through the respective six items of Portuguese Rescue Mission Firefighters—Professional Demands Scale [39] (e.g., ‘Lacking human resources to deal with an occurrence’; α = 0.74). Items were scored on a five-point scale ranging from 1 (rarely) to 5 (very often).

Social support from colleagues. Social support from colleagues was measured through the Job Content Questionnaire [65]. Social support from colleagues was measured with five items (e.g., ‘People I work with are competent in doing their jobs’; α = 0.83). Participants were asked to indicate the extent to which they agreed with each statement on a four-point scale ranging from 1 (strongly disagree) to 4 (strongly agree).

Burnout was measured using the two core dimensions, i.e., emotional exhaustion and cynicism subscales of the Maslach Burnout Inventory-General Survey (MBI-GS) [66]. Emotional exhaustion was measured with five items (e.g., ‘I feel emotionally drained from my job’; α = 0.89), and cynicism with five items (e.g., ‘I doubt the significance of my work’). However, the inspection of factor loadings in the results of the CFA hinted at the elimination of two items from the cynicism scale, due to low factor loadings (i.e., ‘When I am working, I do not like to be bothered with other things’ and ‘I have become more cynical about whether my work contributes anything’), as referred to in previous literature [67]. Thus, with three items, the Cronbach’s alpha of the cynicism scale was 0.76. Participants were asked to rate the frequency of each statement on a seven-point scale ranging from 0 (never) to 6 (every day).

Work engagement was measured using the two core dimensions, i.e., vigor and dedication subscales of the Utrecht Work Engagement Scale general version [31]. Vigor was measured with six items (e.g., ‘At my job, I feel strong and vigorous’; α = 0.75), and dedication with five items (e.g., ‘I am enthusiastic about my job’; α = 0.81). Participants were asked to rate the frequency of each statement on a seven-point scale ranging from 0 (never) to 6 (every day). Previous research has shown that vigor and dedication show a high correlation. Despite this, previous research considers that (1) engagement is a multidimensional construct, (2) vigor and dedication are the core dimensions, and (3) that they are two related but different dimensions [31].

Control variables. In addition to typical demographic variables (i.e., gender, age, and education), specific firefighter variables were also assessed, namely rank in the force, and years of experience as a firefighter, since previous research has shown that they influence well-being in this work population [68,69,70,71]. Fire brigade size was also included as a control variable due to dispersion on the current sample at the fire brigade level (SD = 20.57). 

### 2.3. Data Analyses 

#### 2.3.1. Aggregation Indices

Organization-level demands and social support from colleagues were included as predictors at the second fire-brigade level of analysis. Firefighters’ agreement was assessed using a twofold approach: following a consistency-based approach, both ICC1 and ICC2 indices were calculated. Although there is no fixed cut-off point for ICC, a value of 0.01 might be considered a small effect, a value of 0.10 might be considered a medium effect, and values above 0.25 might be considered a large effect [72]. For the ICC2, values greater than 0.60 support aggregation [73]. The Average Deviation Index (ADM(J)) [74] was computed following a consensus-based approach, whereby fire-brigade agreement was concluded when ADM(J) was equal to or less than 1 [74]. Finally, Analyses of Variance (ANOVA) were computed in order to ascertain whether there was significant between-group discrimination for the measures.

ICC1, ICC2, and ADM(J) indices ranged from 0.10 to 0.16, from 0.70 to 0.77, and from 0.30 to 0.52, respectively. One-way ANOVA results showed statistically significant between-group discrimination for organization-level demands, F(69, 1417) = 3.30, *p* < 0.001; and social support from colleagues, F(69, 1417) = 3.43, *p* < 0.001. By implication, there was a significant degree of between-group discrimination, and therefore the validity of organization-level demands and social support was supported. In conclusion, overall aggregation results indicated within-group agreement in the fire brigades so that firefighters’ perceptions could be aggregated.

#### 2.3.2. Testing the Adequacy of Hierarchical Linear Modeling

ICC was also computed for the case of the dependent variables of the study, that is, the core dimensions of burnout (i.e., emotional exhaustion and cynicism) and the core dimensions of work engagement (i.e., vigor and dedication). In this case, ICC is interpreted as a measure of non-independence, and tests the percentage of variance explained by the aggregated fire-brigade level on the dependent variables, thereby indicating the adequacy of testing hierarchical linear models [75]. 

Non-independence ICC was calculated by conducting an ANOVA model within the general hierarchical linear modeling procedure, which allows the variance to be partitioned for the levels involved in the analyses [76]. ICC results for the dependent variables ranged from 0.02 to 0.09. Although there is no general rule of thumb, results resemble those reported by Bliese [77] with data gathered from the army, which ranged from 0.05 to 0.20. For each outcome variable, variability across the 70 fire brigade intercepts was also examined based on results for the random part in the baseline model. Significant random level-2 intercept coefficients indicate that there is enough variability to include organizational level predictors. Wald’s *t* tests for the level-2 intercept coefficients were significant for emotional exhaustion (τ00 = 0.21; *p* < 0.001), cynicism (τ00 = 0.09; *p* < 0.001), vigor (τ00 = 0.02; *p* = 0.05), and dedication (τ00 = 0.01; *p* < 0.05). Hence, fire brigades differed in terms of intercepts for the dependent variables, thereby allowing tests for cross-level hypotheses.

#### 2.3.3. Hierarchical Linear Models

In order to test the hypotheses, we conducted hierarchical linear modeling (also known as random coefficient modeling) [78] using LISREL 8.8 [79]. Three different hierarchical linear models were tested in a step-by-step approach using maximum likelihood. First, we implemented a random-coefficient regression model (Model 1) in which random coefficients for intercepts and slopes are allowed to fluctuate freely in the baseline equation. Individual-level predictors (i.e., individual-level control variables as well as acute demands and proactive coping) are also included in the equation. This model provides tests of Hypotheses 1a and 1b (main effects), and 1c (interaction effect). The second model, or intercepts-as-outcomes model (Model 2), included fire brigade predictors in the level 2 equation for the intercept (i.e., fire brigade control variables as well as organization-level demands and social support from colleagues). This model allows cross-level effects as stated in Hypotheses 2a and 2b to be tested. This procedure was repeated for each of the four dependent variables, namely emotional exhaustion, cynicism, vigor, and dedication. 

#### 2.3.4. Centering Predictors

For the random-coefficient regression model, individual predictors were grand-mean centered, and their intercepts and slopes were allowed to vary across the fire brigades. Under grand-mean centering, the variance in the intercept term is an adjusted estimator of the between-groups variance in the outcomes as it controls for the individual predictors [80]. For the second model, involving tests of cross-level relationships, fire brigade predictors were also grand-mean centered as this facilitates model estimation [75,77] and alleviates estimation problems at the aggregated level of analysis [81,82].

## 3. Results

### 3.1. Descriptives

Means, standard deviations, internal consistencies, and correlations among the variables in the study are presented in Table 1. As expected, the core dimensions of work engagement (i.e., vigor and dedication) were positively and significantly interrelated, r = 0.84, *p* < 0.001. Similarly, the core dimensions of burnout (i.e., emotional exhaustion and cynicism) were positively and significantly related, r = 0.61, *p* < 0.001. Acute demands were significantly related to organizational demands, r = 0.56, *p* < 0.001, whereas proactive coping was not significantly related to support from colleagues, r = 0.01, *p* > 0.05. 

### 3.2. Hierarchical Regression Analyses

Tests of individual drivers of firefighter’s burnout and work engagement.

Table 2 and Table 3 include results for the hierarchical linear models predicting burnout and work engagement, respectively. Model 1 includes results for Hypotheses 1a and 1b since only firefighter-level predictors were included in the equation along with individual level control variables (i.e., general demographics and specific control variables). 

Following Hypothesis 1a, acute demands were positively and significantly related to both burnout dimensions (β = 0.31, *p* < 0.001, and β = 0.14, *p* < 0.05, for emotional exhaustion and cynicism, respectively). In turn, acute demands were negatively and significantly related to vigor (β = −0.08, *p* < 0.05), whereas they were not related to dedication (β = −0.02, *p* > 0.05). Thus, results provided partial support for Hypothesis 1a. Acute demands were related to emotional exhaustion, cynicism, and vigor, but they were not related to dedication. 

Following Hypothesis 1b, results showed that proactive coping strategies were negatively and significantly related to the burnout dimensions (β = −0.43, *p* < 0.001, and β = −0.53, *p* < 0.001, for emotional exhaustion and cynicism, respectively). For the case of work engagement, proactive coping strategies were positively and significantly related to both work engagement dimensions (β = 0.67, *p* < 0.001, and β = 0.54, *p* < 0.001, for vigor and dedication, respectively). Thus, results provided support for Hypothesis 1b. Proactive coping strategies were negatively related to burnout and positively related to work engagement.

**Figure 2:**
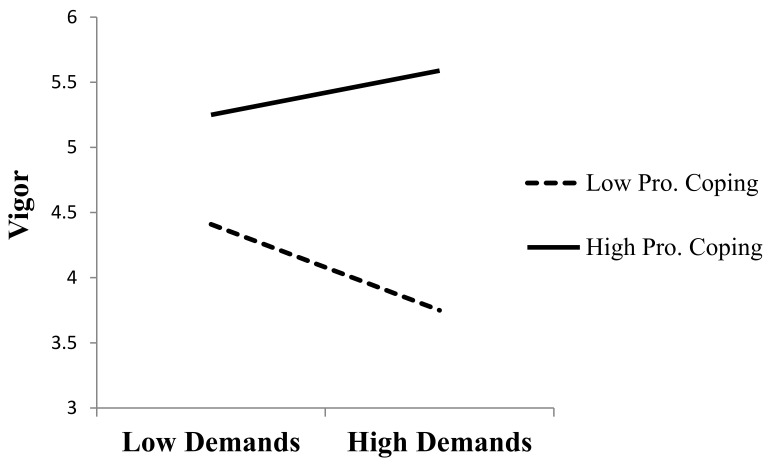
Interaction effects of proactive coping over the relationship between acute demands and vigor.

Results on Hypothesis 1c are included in Table 2 and Table 3 for burnout and work engagement core dimensions, respectively. Results showed that proactive coping strategies were a positive and significant moderator in the case of vigor (β = 0.25, *p* < 0.01). This interaction effect was not significant for the case of dedication, and for the core dimensions of burnout, emotional exhaustion, and cynicism. Hence, proactive coping strategies moderated the effect of acute demands on the firefighters’ vigor, so that acute demands were not depleting energy for those firefighters who were high in proactive coping strategies. Figure 2 depicts the interaction effect of proactive coping strategies in the relationship between acute demands and vigor.

In order to assess the relative importance of individual level predictors over each outcome, we also calculated the proportion of explained variance [82]. To compute these estimates, first we computed the random-coefficient regression model (model 2) fixing slopes for the individual level predictors. Then, we compared the resulting variance estimates with the ones from the ANOVA baseline model. The proportions of explained variance for the outcome variables were 5% (emotional exhaustion), 4% (cynicism), 11% (vigor), and 9% (dedication). 

Tests of organizational drivers of firefighters’ burnout and work engagement.

Hypotheses 2a and 2b dealt with predictors at the organizational, fire brigade level of analysis. Fire brigade size was included as a control variable (see Table 2 and Table 3 for results on burnout and work engagement, respectively). Model 2 yields results for Hypotheses 2a and 2b since it includes organizational-level variables in order to test for cross-level effects. Following Hypothesis 2a, results show that organization-level demands were positively and significantly related to both burnout dimensions (β = 0.78, *p* < 0.01, and β = 0.77, *p* < 0.001, for emotional exhaustion and cynicism, respectively). For the case of work engagement, results indicate that these demands were negatively related to both work engagement dimensions (β = −0.28, *p* < 0.05 and β = −0.30, *p* < 0.01, for vigor and dedication, respectively). Hence, results provided support for Hypothesis 2a. 

Following Hypothesis 2b, results showed that, unexpectedly, social support from colleagues was not significantly related to burnout (β = −0.39, *p* > 0.05, and β = −0.37, *p* > 0.05, for emotional exhaustion and cynicism, respectively). Similarly, social support from colleagues was not significantly related to the two work engagement dimensions (β = 0.31, *p* > 0.05, and β = 0.13, *p* > 0.05, for vigor and dedication, respectively). Thus, Hypothesis 2b was not supported.

The proportion of explained variance was also calculated for each outcome for Model 2 as compared with a model including only firefighter-level predictors (Model 1). The proportions of explained variance between fire brigades (level-2 variance) due to Model 2 were 45% (emotional exhaustion), 43% (cynicism), 60% (vigor), and 50% (dedication).

## 4. Discussion

Using the JD-R Model as a theoretical framework, in the present study we analyzed the relationship between organization-level demands and resources, and individual well-being (i.e., burnout and work engagement) over and above the relationship of individual drivers (i.e., acute demands and proactive coping strategies) in a sample of Portuguese rescue mission firefighters. Furthermore, we explored the moderating role of proactive coping on the relationship between acute demands and individual well-being. Results indicated that proactive coping was related to lower burnout and higher work engagement, whereas acute demands were related to higher burnout and lower work engagement (but only for vigor). Moreover, proactive coping moderated the relationship between acute demands and vigor. Finally, organization-level demands were related to higher burnout and lower work engagement whereas, unexpectedly, social support from colleagues was not related to firefighters’ well-being. The results are unique in the study of well-being by combining measures at different levels, tested by multilevel analyses, and in a specific sample: firefighters. 

### 4.1. Theoretical Implications

Considering the results, further refinement and suggestions for improvement to the JD-R are presented. First, it is necessary to foster a multilevel perspective of this model through a suitable conceptualization of demands and resources at the appropriate level of analysis [21]. Although many studies have tested the role of various types of organizational demands and resources previously [83], an adequate conceptualization to a higher level of analysis shows different relationships to those found when measuring and analyzing demands and resources at the individual level [76]. Based on the seminal work of Demerouti et al. [6], we proposed a definition and conceptualization of organizational demands and resources based on the different processes involved in turning these organizational characteristics into either a shared stressor or a shared resource. That is, in the current context, organization-level demands are fire brigade characteristics that put a similar level of strain on all the firefighting personnel, whereas organizational resources can either be part of systematic organization planning (e.g., human resource practices) or emerge from the interaction and shared experiences of the employees (e.g., social support). Thus, while organizational demands constitute a top-down process, organizational resources may turn into either a top-down process or a bottom-up process [75]. Hence, following a multilevel approach would help to refine the results obtained so far by the extensive body of literature that has emerged in the last decade that has the JD-R as the underlying theoretical model [1,8].

At the individual level of analysis, acute demands showed a significant positive relation with firefighters’ emotional exhaustion and cynicism, while they were negatively and significantly related to firefighters’ vigor, but not related to dedication. Although these findings fully supported previous research on burnout syndrome, the role of firefighters’ acute demands over work engagement differed depending on the dimension involved. Thus, although acute demands became hinder demands depleting employees’ vigor (an energetic and behavioral component of work engagement) [84], they showed no effect on dedication (an emotional dimension of affective attachment toward work). A rationale for this finding may be found in the specific characteristics of the job of firefighters and their role as providers of help, which endows this work population with a strong sense of purpose and meaning toward their job [85]. In fact, in the current sample, firefighters showed a very high average level of dedication (X = 5.35, SD = 0.76) and, in contrast, at the other extreme, a very low level of cynicism (X = 0.93, SD = 1.30). This finding supports the view of a detailed analysis of work engagement by exploring different antecedents and outcomes for its inner components [13,35].

Nevertheless, contrary to expectations, social support was not significantly related to any of the four dimensions of psychological well-being included in the current study. This unexpected finding may be due to the type of relationships that exist in an organizational structure such as fire brigades. Firefighters develop their work in a paramilitary structure and command chain that may yield the salience of coercive leadership and threaten punishments instead of promoting the display of supportive behaviors. Furthermore, firefighting is a male-dominated occupation. In these job settings, males feel more effective when taking an avoidance strategy from adverse situations in daily work [86], whereas women reported seeking social support as more effective [87]. Therefore, in these environments the emotional aspect of work may be granted little importance or employees may develop rules of emotional expression that punish behaviors (i.e., asking for social support) that do not correspond to their expected social role [88]. In fact, the same non-significant result between social support and individual well-being has been found previously in the context of firefighters. Regehr, Hill, Knott, and Sault [89] compared new recruits in the first week of employment and following a 10-week training period with experienced firefighters. In their discussion of results, the authors suggested that opportunities for promotion are limited which breeds competition within tasks more than cooperation and support. 

### 4.2. Practical Implications

Psychological well-being not only depends on the actions taken by the firefighters themselves but also on all the practices and policies that the organization may implement [59]. Although the effect of acute demands is inherent to firefighters’ duties, the development of a proactive coping style may be beneficial for future emergency events. Proactive coping can be trained through interventions focused on effective management of stress [90], as well as interventions aimed at improving goal setting and resource accumulation before the stressful situation arises [47]. The accumulation of resources can lead to positive spirals between well-being and job resources to cope with future stressors [91].

Organization-level demands can be influenced by human resource managers within each fire brigade. Coordination with other security forces is also a task of the coordinators or managers of the brigade and is a source of stress detected in this investigation. All these tasks are not under the control of most firefighters, as they are pushed to focus their full attention on the action at hand, and they thus develop a feeling of lack of control that works at the expense of their psychosocial well-being. The organizational context is a crucial factor in enhancing the possibility of developing interventions that focus on both the individual and the organization as a whole following the conclusions drawn in this study. 

### 4.3. Limitations and Future Research

A major limitation of this study is its cross-sectional nature. This approach cannot determine cause and effect relationships between variables. However, in line with current recommendations in this field, the relations under study did not rely on a single level of analysis but incorporated predictors and covariates at the fire brigade level of analysis. This decision contributed to the expansion of the JD-R Model following a multilevel perspective [13,14,16]. Moreover, we also made use of measures of well-being only obtained through self-report questionnaires that were filled out by the firefighters themselves. We took this approach because our focus of interest was on the employees’ perceptions about their workplace and how these perceptions affect their subjective psychological well-being. Consequently, the common variance bias could be influencing the results [92]. However, in future studies will be interesting to include another source of information for example, the perceptions of key agents: supervisors, colleagues. In addition, we made use of a convenience sample. Despite this, (1) we conducted the analyses in a wide sample of firefighters from all the districts of Portugal, and (2) the representativeness of the answers was maximized. Consequently, it suggested the results could be generalized for this occupational sample. Finally, and despite the high correlation between vigor and dedication, we studied both as outcomes. We did that since previous research has shown that: (1) engagement is a multidimensional construct, (2) vigor and dedication are the core dimensions, (3) both show a high correlation, and despite this, (4) vigor and dedication are considered two related but different constructs [31,93,94].

The current results provide some insight into the processes involved in firefighters’ well-being, but future research may fine-tune our conclusions. Diary studies are a suitable methodology for gathering detailed information on response time, subjective well-being, or actual well-being through psychophysiological indicators [95]. Moreover, firefighters work mainly in the form of action teams [96], hence, a team-level analysis of the tasks conducted by professionals in emergency situations is highly recommended. A collective point-of-view analyzing work in the context of teams would provide detailed information on the social determinants of well-being in the form of both burnout [88] or work engagement [24,97,98]. 

## 5. Conclusions

In conclusion, our findings suggest the association of predictors at individual (by acute demands and proactive behavior) as well as organizational level (by organizational-level demands and social support) on the core dimensions of burnout and work engagement in a special sample: firefighters. Researchers and practitioners should use these results about the role of organizational and individual drivers to ‘enhance’ psychological well-being in fire brigades. Concretely, this can be achieved by implementing practices and policies to guarantee the relevant conditions for improving the well-being of firefighters, developing coping strategies in a proactive way, and enhancing support from colleagues. 

## Figures and Tables

**Table 1:** Means, standard deviations, Cronbach’s alphas, and intercorrelations among the study variables (n = 1487, k = 70).

Variables	M	SD	1	2	3	4	5	6	7	8	9
1. Acute demands	2.66	0.65	(0.74)	0.32 ***	0.04	−0.10 ***	−0.69 ***	0.20 ***	0.10 ***	−0.04	0.00
2. Org. level demands	2.37	0.70	0.56 ***	(0.74)	0.04	−0.32 ***	−0.47 ***	0.27 ***	0.26 ***	−0.18 ***	−0.18 ***
3. Proactive coping	3.33	0.43	−0.04	0.15 ***	(0.71)	0.04	−0.02	−0.11 ***	−0.16 ***	0.31 ***	0.28 ***
4. Colleague’s support	2.99	0.47	−0.52 ***	−0.59 ***	0.01	(0.83)	0.56 ***	−0.13 ***	−0.17 ***	0.22 ***	0.25 ***
5. Size of fire brigade	21.24	20.57	−0.33 ***	−0.17 ***	-0.01	0.03	(−)	−0.56 ***	−0.33 ***	0.11 ***	0.05 *
6. Emotional exhaustion	2.25	1.52	0.69 ***	0.60 ***	-0.01	−0.58 ***	−0.21 ***	(0.89)	0.49 ***	−0.29 ***	−0.26 ***
7. Cynicism	0.93	1.30	0.46 ***	0.54 ***	−0.06 *	−0.47 ***	−0.10 ***	0.61 ***	(0.76)	−0.31 ***	−0.39 ***
8. Vigor	4.93	0.87	−0.16 ***	−0.35 ***	0.23 ***	0.32 ***	0.03	−0.40 ***	−0.55 ***	(0.75)	0.74 ***
9. Dedication	5.35	0.76	−0.12 ***	−0.35 ***	0.11 ***	0.24 ***	0.01	−0.36 ***	−0.47 ***	0.84 ***	(0.81)

Note. Cronbach’s alphas over the main diagonal. Intercorrelations are presented at the individual level (below the main diagonal; n = 1487) and at the fire brigade level (above the main diagonal; k = 70). * *p* < 0.05. *** *p* < 0.001.

**Table 2:** Results for the hierarchical linear models predicting Emotional Exhaustion and Cynicism.

	Emotional Exhaustion		Cynicism	
Parameters	Model 1	Model 2	Model 1	Model 2
Intercept	3.53 *** (0.43)	3.28 *** (0.42)	1.02 ** (0.37)	0.83 * (0.37)
Level 1 (firefighters)				
Type of firefighter	−0.23 * (0.09)	−0.08 (0.11)	−0.09 (0.07)	−0.02 (0.09)
Gender	−0.01 (0.16)	0.02 (0.16)	0.28 * (0.14)	0.29 * (0.14)
Age	−0.02 * (0.01)	−0.02 * (0.01)	−0.01* (0.01)	−0.01 * (0.01)
Education	−0.08 (0.05)	−0.08 (0.05)	−0.03 (0.03)	−0.02 (0.05)
Rank in the force	−0.04 (0.04)	−0.04 (0.04)	0.02 * (0.01)	−0.03 (0.03)
Years of experience	0.02 * (0.01)	0.02 * (0.01)	0.01 (0.06)	0.02 * (0.01)
Acute demands (AD)	0.31 *** (0.06)	0.29 *** (0.06)	0.14 * (0.06)	0.11 * (0.06)
Proactive coping (PC)	−0.43 *** (0.09)	−0.44 *** (0.09)	−0.53 *** (0.08)	−0.53 *** (0.08)
ADxPC	−0.11 (0.15)	−0.13 (0.15)	0.03 (0.13)	0.01 (0.13)
Level 2 (fire brigades)				
Fire brigade size		−0.00 (0.00)		−0.00 (0.00)
Org.-level demands		0.78 ** (0.24)		0.77 *** (0.21)
Colleagues’ support		−0.39 (0.37)		−0.37 (0.31)

Note. Standard errors are in parentheses. * *p* < 0.05. ** *p* < 0.01. *** *p* < 0.001.

**Table 3:** Results for the hierarchical linear models predicting Vigor and Dedication.

	Vigor		Dedication	
Parameters	Model 1	Model 2	Model 1	Model 2
Intercept	4.73 *** (0.24)	4.86 *** (0.24)	5.32 *** (0.21)	5.42 *** (0.21)
Level 1 (firefighters)				
Type of firefighter	−0.02 (0.04)	−0.08 (0.05)	−0.04 (0.04)	−0.10 * (0.05)
Gender	0.03 (0.09)	0.01 (0.09)	−0.01 (0.08)	−0.03 (0.08)
Age	0.01 ** (0.00)	0.01 ** (0.00)	0.12 ** (0.00)	0.01 ** (0.00)
Education	−0.07 * (0.03)	−0.06 * (0.03)	−0.06 * (0.03)	−0.06 * (0.03)
Rank in the force	0.05 * (0.02)	0.05 * (0.02)	0.01 (0.02)	0.02 (0.02)
Years of experience	−0.01 * (0.01)	−0.01 * (0.01)	−0.01 * (0.01)	−0.01 * (0.00)
Acute demands (AD)	−0.08 * (0.04)	−0.06 (0.04)	−0.02 (0.03)	−0.00 (0.03)
Proactive coping (PC)	0.67 *** (0.05)	0.67 *** (0.05)	0.54 *** (0.05)	0.54 *** (0.05)
ADxPC	0.25 ** (0.09)	0.25 ** (0.09)	−0.01 (0.08)	−0.00 (0.08)
Level 2 (fire brigades)				
Fire brigade size		0.01 (0.02)		0.00 (0.00)
Org.-level demands		−0.28 * (0.12)		−0.30 ** (0.11)
Colleagues’ support		0.31 (0.18)		0.13 (0.17)

Note. Standard errors are in parentheses. * *p* < 0.05. ** *p* < 0.01. *** *p* < 0.001.
